# Retraction: Adsorption and sensor performance of transition metal-decorated zirconium-doped silicon carbide nanotubes for NO_2_ gas application: a computational insight

**DOI:** 10.1039/d5ra90036k

**Published:** 2025-04-07

**Authors:** Ismail O. Amodu, Faith A. Olaojotule, Miracle N. Ogbogu, Oluwatobi A. Olaiya, Innocent Benjamin, Adedapo S. Adeyinka, Hitler Louis

**Affiliations:** a Computational and Bio-Simulation Research Group, University of Calabar Calabar Nigeria louismuzong@gmail.com; b Department of Mathematics, University of Calabar Calabar Nigeria; c Department of Research Analytics, Saveetha Dental College and Hospitals, Saveetha Institute of Medical and Technical Sciences, Saveetha University Chennai India; d Department of Chemical Sciences, University of Johannesburg Pretoria South Africa

## Abstract

Retraction of ‘Adsorption and sensor performance of transition metal-decorated zirconium-doped silicon carbide nanotubes for NO_2_ gas application: a computational insight’ by Ismail O. Amodu *et al.*, *RSC Adv.*, 2024, **14**, 5351–5369, https://doi.org/10.1039/D3RA08796D.

The Royal Society of Chemistry hereby wholly retracts this *RSC Advances* article due to concerns with the reliability of the data.

The molecular dynamic (MD) simulations have not been described satisfactorily with mistakes throughout, such as the misuse of the term “time-step”. In addition, some of the results are inconsistent with the statements made by the authors, such as the temperature used, as well as the data in Table 4 being impossible to obtain from the formulae mentioned by the authors.

The authors have not been able to provide the input or trajectory file generated from the MD simulations.

In addition, the affiliation for the corresponding author was given incorrectly in the original article. This has been corrected in this notice.

Given the significance of these concerns, the Editor has lost confidence that the findings presented in this paper are reliable.

The authors were informed but have not responded to any correspondence regarding the retraction.

Laura Fisher, Executive Editor, *RSC Advances*

28th March 2025

